# Supporting entrepreneurial resilience: An experimental study protocol

**DOI:** 10.1371/journal.pone.0349194

**Published:** 2026-06-04

**Authors:** Kompal Sinha, Nyamdavaa Byambadorj, Elisabetta Magnani, Rong Zhu, Francesco Chirico, Yuanyuan Gu, Roy Green

**Affiliations:** 1 Department of Economics, Macquarie Business School, Macquarie University, Sydney, New South Wales, Australia; 2 Global Labor Organisation (GLO), Essen, Germany; 3 College of Business, Government and Law, Flinders University, Adelaide, Australia; 4 Institute of Labor Economics (IZA), Bonn, Germany; 5 Department of Management, Macquarie University, Sydney, New South Wales, Australia; 6 Macquarie University Centre for Health Economy, Macquarie University, Sydney, New South Wales, Australia; 7 University of Technology Sydney, Sydney, New South Wales, Australia; PLOS: Public Library of Science, UNITED KINGDOM OF GREAT BRITAIN AND NORTHERN IRELAND

## Abstract

Entrepreneurial activity is shaped by the structure and functioning of the entrepreneurial ecosystem, yet there is limited consensus on how entrepreneurial resilience is defined or measured. This study protocol outlines the development of a novel, empirically grounded measure of entrepreneurial resilience, conceptualised as a multidimensional and dynamic capability that enables adaptation under contextual hardship and resource constraints. Guided by a capability-based framework, the study draws on prior literature and employs qualitative methods, including a multi-round Delphi study and stakeholder interviews and focus groups. The study components will identify context-specific mechanisms and translate expert consensus into measurable and policy-relevant indicators. By setting out a transparent and reproducible methodology, this protocol contributes to the entrepreneurship and behavioural economics literature and provides a foundation for future empirical work and targeted policy interventions to support business continuity in volatile environments.

## 1 Introduction

**Fig 1:**
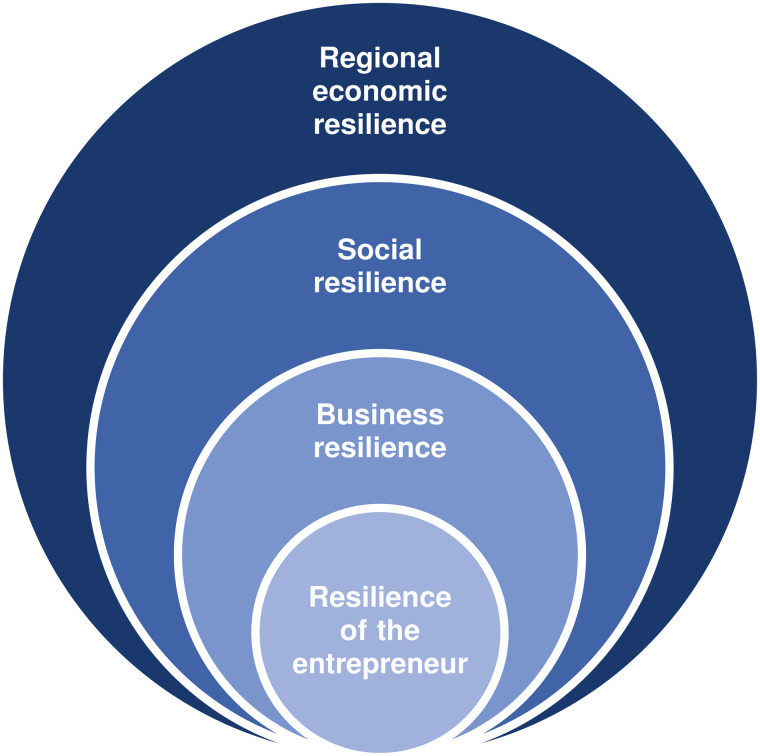
Structure of resilience.

Resilience has long been recognised as a dynamic process through which individuals adapt positively in the face of serious adversity [1]. In the entrepreneurship literature, resilience is frequently used as an umbrella term capturing a wide spectrum of behaviours and adaptive responses [2]. It has been associated with the ability to go on with life after adversity [3] and bounce back from a crisis [4]. Although the resilience of entrepreneurs is at the core of the concept of resilience (Fig 1) [5], the existing literature has largely focused on resilience at the regional, organisational, or societal levels. This focus implicitly assumes that entrepreneurial resilience emerges naturally without adequately accounting for the micro-level characteristics, lived experiences, and life-course conditions that shape individual entrepreneurs’ capacity to respond to adversity [2]. As a result, the concept of entrepreneurial resilience remains comparatively underdeveloped [5].

While the extant literature has developed measures of resilience such as the Connor-Davidson Resilience Scale (CDRISC) [6], the measures are static in nature and do not account for the dynamics of life experiences in influencing resilience. Moreover, the CD-RISC scale does not account for socioeconomic circumstances, major life events or family dynamics, their duration and severity in a range of personal and professional domains of life. From a policy perspective, this is important limitation, as resilience depends on how challenges develop across time and place and influence entrepreneurial survival. Distinguishing between spatially transient resilience and persistent resilience is crucial for designing targeted interventions. Entrepreneurs who maintain resilience despite recurring hardship may require a different policy approach than those who cycle in and out of resilience or experience frequent interruptions in their adaptive capacity. Drawing on a capability-based approach [7,8], we conceptualise entrepreneurial resilience as a multidimensional construct shaped by: (1) the breadth of hardships experienced across multiple domains within a given period; (2) the duration of hardships within specific domains across multiple years; and (3) the chronicity of hardships, defined as uninterrupted spells of adversity over time. This life-course perspective acknowledges that personality traits may provide an initial foundation for resilience, but situational circumstances—economic instability, health shocks, caregiving responsibilities, or repeated financial strain—can erode or strengthen resilience in different ways. In this context, resilience loss is not presumed to follow a uniform pattern. Individuals experiencing hardship intermittently (for example, every second year) may have the emotional, financial, or social resources to recover and continue operating their businesses. By contrast, entrepreneurs facing continuous and uninterrupted hardship are more likely to experience cumulative disadvantage, diminished resilience, and heightened risk of poor business decisions. Capturing these differences is critical for tailoring policies to sub-groups of entrepreneurs, especially those vulnerable to falling into a persistent low-resilience state.

This study seeks to address these conceptual and empirical gaps by developing a multidimensional measure of entrepreneurial resilience. The development of this protocol was informed by insights from prior systematic reviews of the literature, which helped identify conceptual gaps and measurement challenges in the field. To achieve this, we employ a sequential mixed-methods design that integrates a multi-round Delphi study, stakeholder in-depth interviews and focus groups, and a discrete choice experiment (DCE). This approach enables the identification, prioritisation, and quantification of the drivers, drainers, and policy levers associated with entrepreneurial resilience across different contexts.

The paper is organised as follows. Section 2 outlines the methodology, describing the sequential mixed-methods design comprising Delphi study, focus groups, and discrete choice experiment (DCE), along with participant recruitment, procedures, analytical strategy, and ethical considerations. Section 3 summarises the expected outcomes and anticipated contributions of the research and concludes the paper.

## 2 Methodology

### 2.1 Study design

To analyse the grand challenge of measuring entrepreneurial resilience and identifying the factors that drive or drain resilience among entrepreneurs, we employ a sequential mixed-methods design comprising three interlinked phases: (1) a Delphi study, (2) in-depth interviews and focus groups, and (3) a discrete choice experiment (DCE). Each phase builds cumulatively on the previous one, integrating conceptual, contextual, and empirical insights to develop a coherent and policy-relevant resilience framework. The development of the study instruments was informed by prior reviews of the literature and existing theoretical work on resilience. Delphi surveys will be administered online and will follow a five-stage structure, beginning with an internal Round 0 used to scope and refine the initial item bank based on prior literature and pilot feedback from 4–6 advisors. Subsequent survey rounds (Rounds 1–4) will combine pre-defined and emergent questions to iteratively build expert consensus. Across all rounds, participants will rate items on a 7-point Likert scale for importance and feasibility, with optional measurability, following established Delphi guidelines [9–13]. Reporting of the Delphi and qualitative components is informed by established guidance, including the CREDES Delphi reporting standard [14] and the COREQ checklist for qualitative research [15]. See Appendix A for details.

Interviews and focus groups add contextual nuance, examining how the prioritised drivers and drainers from the Delphi manifest across settings such as gender, digitalisation, and exposure to shocks (e.g., COVID-19, conflict, or economic crises). Focus groups will be conducted virtually (e.g., Zoom or Microsoft Teams), audio-recorded, and transcribed, with stratified group composition (e.g., youth, women, high/low digital intensity, shock exposure) to surface heterogeneous mechanisms and dual-role factors (such as institutions acting as both enablers and barriers).

Finally, insights from both the Delphi and the qualitative phase will be synthesised to generate and refine the attributes and levels for the DCE. The DCE will be implemented online (SurveyEngine) to quantify stakeholder preferences and trade-offs over specific interventions and policy levers that strengthen entrepreneurial resilience (e.g., mentoring and coaching, liquidity grants, digital enablement packages, streamlined policy processes). Together, these phases culminate in an integrated set of outputs: a consensus-based conceptual framework of entrepreneurial resilience, a prioritised list of drivers and drainers, and a pilot-ready DCE instrument that operationalises these constructs into measurable, policy-relevant indicators.

Participant recruitment has not yet commenced. Recruitment is anticipated to begin in the first quarter of 2026, with data collection expected to occur during second quarter of 2026. Data analysis and dissemination of results are anticipated in the third quarter of 2026.

### 2.2 Delphi methodology

The extant literature on entrepreneurial resilience is highly fragmented across contexts—including COVID-19 shocks, conflict environments, psychological resources, institutional settings, social capital, digital capability, and financial constraints—resulting in substantial conceptual and measurement heterogeneity. As prior studies in entrepreneurship and management have shown [16,17], the Delphi method is well suited for resolving such heterogeneity by building expert consensus on contested constructs. A structured Delphi is therefore required to establish shared definitions of entrepreneurial resilience and to prioritise drivers, drainers, and outcome indicators before empirical measurement can proceed.

A second source of inconsistency arises from methodological variation: existing studies range from conceptual papers and qualitative interviews to psychometric scales, econometric analyses, and policy evaluations. This pluralism produces incompatible metrics and a lack of agreement on outcomes such as continuity, time-to-recovery, liquidity access, or digital capability. A Delphi enables convergence on a core measurement toolkit grounded in expert judgement [12,18].

Prior literature highlights that some factors (e.g., institutions) may act as both drivers and drainers depending on context. A Delphi with explicit context tagging (e.g., COVID, conflict/fragility, gendered, youth, digital) allows experts to clarify when and why the same factor contributes to or detracts from resilience.

Following established Delphi guidelines [9–11,19], our study uses Round 0 to scope, refine, and pilot the initial item bank derived from prior literature and theoretical work, including de-duplication and consolidation of overlapping indicators. Rounds 1–4 combine pre-defined and emergent questions to iteratively build consensus on: (a) the definition and scope of entrepreneurial resilience; (b) priority drivers and drainers across contexts; (c) appropriate measurement indicators and outcome metrics; and (d) policy and program levers that strengthen resilience. Across all rounds, experts rate items on a 7-point Likert scale for importance and feasibility, with optional measurability [12,13]. The qualitative phase complements the Delphi by illuminating mechanisms and lived pathways underlying the ranked items—particularly in domains influenced by gender, digital divides, conflict exposure, or resource scarcity. Focus groups provide causal detail and contextual nuance that consensus ratings alone cannot capture. Together, the Delphi and qualitative analysis create a triangulated foundation [20] for developing and validating the resilience framework and the subsequent DCE attributes.

#### 2.2.1 Delphi panel recruitment and participant characteristics.

Following recommended protocols for Delphi studies, we will purposively recruit domain experts with demonstrated expertise in entrepreneurship, innovation, and resilience. Eligibility required active engagement in at least one of the following areas: academic research on entrepreneurship or resilience, entrepreneurial practice, ecosystem leadership (e.g., incubators, accelerators, investor networks), or policymaking related to SMEs. Experts will be identified through publications on entrepreneurial resilience, professional and policy networks, and targeted outreach to ecosystem organisations. Recruitment will aim to ensure geographic diversity across OECD regions (e.g., USA, Germany, and Australia).

The ideal Delphi sample size ranges from 15 to 23 participants [21]; however, for a multistakeholder analysis such as this, a larger panel improves replicability and diversity of perspectives [22]. We therefore aim for a panel of approximately 70–85 respondents, representing non-OECD contexts, gender diversity, institutional roles, and social dimensions highlighted in prior literature. We will aim for diversity in gender, geography, and disciplinary background, including experts from business, economics, public health, and related fields. Given typical attrition rates of 15%–40% in Delphi studies [21,23], we will recruit approximately 100–120 experts in Round 1, with the aim of retaining around 70–85 participants by Round 4.

#### 2.2.2 Survey development and questions.

Survey questions were developed in alignment with previous Delphi frameworks [10] and qualitative research guidelines [24,25]. Questionnaires combine closed-ended Likert-type items with open-ended questions to elicit both structured ratings and richer qualitative justification.

- Round 1 will focus on conceptual foundations and initial item generation. Participants will first provide background information and an open-ended definition of entrepreneurial resilience, specifying what should be considered inside and outside the construct. They will then list up to five key drivers and five key drainers of resilience based on their experience, before reviewing a literature-informed category list and indicating any additional factors they regard as important.
- Building on synthesised Round 1 responses, Round 2 will ask participants to rate the shortlisted drivers and drainers on (i) their importance for entrepreneurial resilience and (ii) their actionability through policy, training, or support programs, using Likert-type scales. For each factor, respondents will also indicate whether it is context-sensitive (e.g., COVID-19, gender, youth, digital transformation, conflict, senior entrepreneurs), enabling us to tag items by setting and population. Free-text fields will allow participants to suggest additional drivers, drainers, or contextual notes.
- Round 3 will shift to measurement and outcomes. For each priority factor, participants will select preferred measurement approaches (e.g., validated psychometric scales, longitudinal surveys, theoretical or econometric modelling, mixed-methods designs) and may recommend additional methods. They will then choose and rank outcome metrics for entrepreneurial resilience (such as business survival/continuity, growth, adaptability, time-to-recovery, access to liquidity, digital capability indices, and workforce resilience), identifying those most relevant for policy and program design.
- In Round 4, participants will review a consolidated list of drivers and drainers derived from Rounds 1–3 and will indicate their level of agreement with including each in the final framework. They will also rate the importance and feasibility of key policy and program levers (e.g., startup funding, relief and liquidity access, digital infrastructure, mentoring and coaching, gender-inclusive policies) and will rank their top three priorities. A final open-ended question will invite additional recommendations on measurement tools, outcome metrics, and resilience-building strategies.

All Delphi rounds will be administered online using SurveyEngine, with each questionnaire designed to take approximately 15–20 minutes and to permit asynchronous completion. Full survey instruments for Rounds 1–4 are provided in Appendix B.

#### 2.2.3 Data analysis.

Consensus will be assessed using a combination of central tendency and dispersion criteria, alongside Kendall’s coefficient of concordance (*W*) [26]. An item will be considered to have reached consensus if it achieves a median rating of ≥6 on the 7-point Likert scale and an IQR ≤1. Stability across successive rounds will be evaluated by examining changes in medians, IQRs, and Kendall’s *W*; items showing minimal change between rounds will be treated as stable, while unstable items will be re-presented or refined in subsequent rounds. Subgroup comparisons (e.g., by region or professional role) will be undertaken where relevant. Open-ended responses will be coded using an *a priori* codebook derived from prior literature and the study’s conceptual framework, with allowance for emergent themes. Two coders will independently code responses; inter-rater reliability will be assessed using Cohen’s κ [27]. Discrepancies will be resolved through discussion and adjudication. Iterative synthesis across rounds will track movement toward consensus.

### 2.3 Interviews and focus groups

In-depth interviews and focus groups are designed to complement the Delphi study by examining how prioritised drivers and drainers of entrepreneurial resilience operate in practice, and by exploring the mechanisms through which these factors enable or constrain entrepreneurs in different contexts. Whereas the Delphi process elicits structured expert consensus, the focus groups will generate context-rich narratives that illuminate dual-role factors (e.g., institutions acting as both enablers and barriers) and inform the design and wording of DCE attributes and levels [28]. In-depth interviews are expected to be the primary qualitative method, as they allow deeper, more considered individual reflections, while focus groups may be used where interactive discussion is beneficial.

#### 2.3.1 Sampling and recruitment.

We will use purposive sampling [29] to recruit entrepreneurs and ecosystem actors whose experiences reflect the contextual lenses identified in prior literature and the Delphi rounds, including gender, age (youth versus senior entrepreneurs), digital intensity, and exposure to major shocks such as COVID-19 or conflict-related disruptions. Participants will be identified through business networks, incubators, professional associations, and existing project contacts, with limited snowball sampling to reach under-represented groups. We anticipate conducting approximately 15–25 in-depth interviews and, where appropriate, 4–6 small focus groups with 5–8 participants each. Qualitative formats will be selected based on feasibility, participant availability, and the suitability of the topic for individual versus group discussion. Stratification (e.g., by gender, age, digital intensity, or exposure to major shocks) will be used to capture variation in how drivers and drainers of resilience are experienced across contexts.

#### 2.3.2 Data collection procedures.

In-depth interviews and focus groups will be conducted virtually (e.g., via Zoom or Microsoft Teams) to enable participation across regions and accommodate entrepreneurial schedules. Interviews are expected to last approximately 20–40 minutes and focus groups 50–70 minutes. Sessions will be facilitated by experienced qualitative researchers. With participants’ consent, discussions will be audio-recorded and transcribed verbatim. A semi-structured topic guide, informed by prior literature and Delphi findings, will ensure coverage of key domains while allowing for emergent issues [30].

Participants will be invited to describe recent adverse events or periods of uncertainty and their responses. Facilitators will use Delphi-derived prompts (e.g., self-efficacy, social support, institutional trust, digital readiness, financial buffers, gender norms) to explore which factors were most influential in their trajectories. Where focus groups are conducted, brief participatory exercises (such as card-sorting and scenario prioritisation) may be used to guide refinement of DCE attributes.

#### 2.3.3 Link to analysis and DCE design.

Interview and focus-group transcripts will be analysed using the same directed content analysis approach applied to open-ended Delphi responses [31]. Coding will draw on the Delphi-derived codebook, with additional inductive codes created where new mechanisms or contextual nuances emerge. Two researchers will code transcripts independently using qualitative analysis software (e.g., NVivo), and inter-rater reliability will be assessed using Cohen’s κ [27]. Coding discrepancies will be resolved through discussion and consensus; where disagreement persists, a third senior researcher will adjudicate. Thematic matrices will be used to compare factors across groups and to identify convergent, complementary, or divergent patterns.

These qualitative findings will play a dual role: (a) deepening understanding of how drivers and drainers of entrepreneurial resilience operate in practice, including instances where the same factor functions as both an enabler and a constraint; and (b) informing the refinement of DCE attributes and levels to ensure that the experimental design reflects realistic, interpretable, and policy-relevant trade-offs for entrepreneurs.

### 2.4 Discrete choice experiment (DCE)

The final phase of the study will employ a discrete choice experiment (DCE) to quantify stakeholder preferences and trade-offs over specific policy and program levers that support entrepreneurial resilience. Whereas the Delphi and the qualitative phase establish what matters and why, the DCE will estimate the relative importance of different support attributes and the willingness of entrepreneurs to trade one form of support for another under resource constraints.

DCE attributes and levels will be developed iteratively from three inputs: (1) Delphi Round 4 priorities on policy and program levers, (2) qualitative card-sorting and scenario prioritisation exercises conducted during interviews and focus groups, and (3) thematic domains highlighted in prior literature on entrepreneurial resilience. Candidate attributes will reflect the main levers highlighted across these sources, for example:

- **Type of support** (e.g., psychological, peer support, mentoring/coaching, liquidity or relief grant, digital infrastructure and skills package, policy fast-track or regulatory streamlining);
- **Access frictions** (e.g., documentation burden, approval time, reporting requirements), capturing the role of bureaucracy versus streamlined processes; procurement preferences, marketplace platform access, export facilitation, capturing the demand side access challenges.
- **Targeting transparency, and inclusion** (e.g., universal programs versus schemes prioritising women, youth, or shock-affected entrepreneurs);
- **Financial security infrastructure** (e.g., size and duration of grants, availability of emergency buffers or contingent support, failure and recovery);
- **Digital enablement** (e.g., connectivity, hardware/software access, tailored digital skills training, building artificial intelligence-specific capability).

Attributes will be refined through an internal workshop with the research team and advisory group to ensure that (a) they map cleanly onto the resilience domains (psychological, social, institutional/policy, economic/financial, digital, gender/youth), (b) levels are realistic and policy-relevant, and (c) the overall design remains cognitively manageable for respondents. A small pre-test (cognitive interviews with 8–10 entrepreneurs) will be conducted to check comprehension, perceived realism, and the clarity of attribute descriptions and choice tasks; wording and levels will be adjusted accordingly. These cognitive interviews will also provide qualitative feedback on how respondents interpret resilience-related support mechanisms.

#### 2.4.1 Experimental design and choice tasks.

The DCE will be administered online using SurveyEngine. Each choice task will present respondents with two hypothetical policy support alternatives and a status quo (no additional support) option. Respondents will be asked to select their preferred option in each task. A D-efficient experimental design will be generated using NGene software. Initial priors will be obtained from the pilot survey and qualitative insights from the Delphi and qualitative phases. These priors will be updated following pilot estimation to refine the final experimental design for the main survey. The number of choice tasks per respondent will be limited to maintain cognitive feasibility (approximately 8–12 tasks), with blocking applied where necessary. Attributes will be coded using effects coding to facilitate interpretation and avoid confounding with the intercept. The design will ensure level balance and minimal correlation across attributes, while allowing estimation of key main effects and a limited set of theoretically motivated interactions (e.g., type of support by targeting).

#### 2.4.2 Study sample and recruitment.

The DCE sample will consist of entrepreneurs, SME owner–managers, and key ecosystem actors such as incubator managers and investor representatives. SurveyEngine, an international panel provider with experience in recruiting specialised business samples, will administer the survey and manage recruitment across the three countries. Soft quotas will be applied to ensure diversity across gender, age (youth versus senior entrepreneurs), sector, and digital intensity. We aim to recruit approximately 300 respondents per country (total target sample of around 900 participants). This target aligns with established recommendations for estimating mixed logit models and preference heterogeneity in DCEs [32,33]. Final sample targets may be refined based on pre-test response rates, the number of attributes and levels included in the experimental design, and specific model identification requirements.

#### 2.4.3 DCE analysis.

DCE data will be analysed within a random utility maximisation framework [34]. Each choice will be modelled as a function of the attributes and levels presented in the choice tasks. Mixed logit models [35,36] will be used to estimate preference parameters and to account for random taste heterogeneity across respondents. These models will quantify the trade-offs entrepreneurs are willing to make across policy and support characteristics.

From the estimated models, we will derive:

- the relative importance of each attribute in shaping support preferences;
- marginal rates of substitution between key attributes (e.g., mentoring versus liquidity support, or digital enablement versus processing time);
- predicted uptake of alternative policy packages under different design scenarios; and
- subgroup differences in preferences (e.g., by gender, youth status, digital intensity, or exposure to recent shocks).

Robustness checks will include alternative model specifications such as conditional logit or latent class models. Quantitative findings from the DCE will be triangulated with Delphi and qualitative insights from interviews and focus groups to identify which combinations of policy supports are both highly valued and contextually feasible, thereby informing evidence-based interventions to strengthen entrepreneurial resilience.

### 2.5 Ethical considerations

Ethics approval for this study was granted by the Macquarie University Human Research Ethics Committee via the Macquarie Business School Subcommittee (Reference No: 520251972865737; Project ID: 19728) on 27/11/2025. The project (*Supporting Dynamic Multidimensional Entrepreneurial Resilience in Australia*) was approved as meeting the requirements of the *National Statement on Ethical Conduct in Human Research (2023)* [37]. Participation in the Delphi, interviews, focus groups, and DCE components will be voluntary, with electronic informed consent obtained through the SurveyEngine platform. Participants will receive an information sheet outlining study aims, procedures, and confidentiality protections, and may withdraw at any time before data aggregation. All data will be de-identified and stored securely on password-protected servers at Macquarie University, with access restricted to authorised members of the research team. As the study involves expert opinions rather than personal or clinical data, risks to participants are minimal.

## 3 Conclusion

The study will yield a comprehensive framework and empirical evidence on entrepreneurial resilience. Specifically, the Delphi and qualitative analyses (interviews/focus groups) will generate a consensus definition of entrepreneurial resilience, a prioritised list of key drivers and drainers, and recommended measurement indicators. The DCE will quantify preferences for policy and program interventions, revealing which forms of support are most valued and what trade-offs stakeholders are willing to make. These combined findings will inform evidence-based recommendations for policies and programs designed to strengthen entrepreneurial resilience, improve business continuity, and enhance adaptive capacity across diverse economic and social contexts. The integrated analysis is expected to inform the development of a framework for entrepreneurial resilience, integrating expert consensus, qualitative narratives, and quantitative preference data. Together, these results will highlight the most critical factors influencing resilience and identify the most effective strategies for fostering adaptive capacity and sustainability among entrepreneurs. The Delphi and qualitative analyses will uncover the conceptual and contextual foundations of resilience, while the DCE will quantify stakeholder preferences for various policy and program levers. The combined findings will offer evidence-based recommendations for policymakers, educational institutions, and ecosystem organisations on how to design targeted interventions—such as mentoring, liquidity support, or digital enablement—that enhance entrepreneurial resilience in volatile environments. Looking ahead, the research team plans to refine and expand this work by using the Delphi Round 4 and focus group card-sorting results to adjust and validate DCE attributes in subsequent experimental studies. Future phases will include longitudinal or quasi-experimental evaluations to test the operationalisation of resilience-enhancing strategies in real-world entrepreneurial ecosystems. Ultimately, this program of research aims to advance both theory and practice by translating the measurement of entrepreneurial resilience into practical frameworks that support business continuity and innovation over time.

## Supporting information

S1 File This is the supporting file for Appendix Tables. This includes the following tables: Table A1 - Conducting and Reporting Delphi Studies (CREDES) Checklist and Application to this Delphi Study; Table A2 - Consolidated Criteria for Reporting Qualitative Studies (COREQ) and Application to Interviews and Focus Groups; Table B1 - Summary of Delphi Round 1 Questionnaire Items; Table B2 - Summary of Delphi Round 2 Questionnaire Items; Table B3 - Summary of Delphi Round 3 Questionnaire Items; Table B4 - Summary of Delphi Round 4 Questionnaire Items; Table B5 - Interview/focus group topic guide.
(PDF)

S1 File Final manuscript 20 May Tex. (TEX)
