# Supplementary material for: Supporting entrepreneurial resilience: An experimental study protocol
Source: PLoS One. 2026 Jun 4;21(6):e0349194. doi: 10.1371/journal.pone.0349194 (PMC13235934; doi:10.1371/journal.pone.0349194)
Supplement: S1 File — This includes the following tables: Table A1 - Conducting and Reporting Delphi Studies (CREDES) Checklist and Application to this Delphi Study; Table A2 - Consolidated Criteria for Reporting Qualitative Studies (COREQ) and Application to Interviews and Focus Groups; Table B1 - Summary of Delphi Round 1 Questionnaire Items; Table B2 - Summary of Delphi Round 2 Questionnaire Items; Table B3 - Summary of Delphi Round 3 Questionnaire Items; Table B4 - Summary of Delphi Round 4 Questionnaire Items; Table B5 - Interview/focus group topic guide. (PDF) [file pone.0349194.s001.pdf]

Appendices

Appendix A: Checklists

Table A1. Conducting and Reporting Delphi Studies (CREDES) Checklist and Application to this Delphi Study

No.	Criteria	Requirement for Delphi Studies	Application in This Delphi Study
1	Justification	Delphi must be justified as the best method for structured expert input and consensus.	Used to gather and refine views from entrepreneurs, policymakers, investors, and ecosystem actors to prioritise resilience factors.
2	Planning and Process	Modifications must be justified and applied consistently.	3–4 round Delphi: R1 ideas, R2 rating, R3 feedback, R4 prioritisation; all changes documented.
3	Consensus Definition	Consensus rules must be predefined.	Consensus via median, IQR, and % agreement; borderline items revised; low-agreement items removed.
4	Informational Input	Materials must be neutral and pilot-tested.	Lists and measures based on review, piloted with 6–8 advisors, refined for clarity and neutrality.
5	Prevention of Bias	Avoid influencing judgement; ensure anonymity.	Anonymous rounds in SurveyEngine with only statistical, anonymised feedback; no conflicts of interest.
6	Interpretation	Consensus does not equal correctness; divergence matters.	Report agreement, disagreement, and divergence across contexts and ecosystems.
7	External Validation	Final guidance should be externally reviewed.	Indicators and levers reviewed by advisory panel and presented at conferences.
8	Purpose & Rationale	Purpose must justify Delphi as optimal.	Delphi fits multidimensional, cross-country resilience framework across AU/US/DE.
9	Expert Panel	Panel criteria and composition documented.	70-85 experts across three countries: entrepreneurs, investors, advisors, policymakers, academics, lenders.
10	Methods Description	Methods must be fully reported.	Document evidence synthesis, questionnaire design, piloting, platform, rounds, consensus rules, and item revisions.
11	Procedure Flow	Flowchart recommended.	Scoping $\rightarrow$ focus group $\rightarrow$ pilot $\rightarrow$ R1 $\rightarrow$ analysis $\rightarrow$ R2 $\rightarrow$ analysis $\rightarrow$ R3 $\rightarrow$ optional R4 $\rightarrow$ synthesis $\rightarrow$ external review $\rightarrow$ dissemination.
12	Attainment of Consensus	Must state rules for non-consensus items.	Threshold: IQR ≤ 1 and $\geq 70\%$ agreement; borderline revised; non-consensus removed where appropriate.
13	Results Reporting	Each round must be reported separately.	Report distributions, changes, stability, revisions, and final rankings.
14	Limitations	Must acknowledge limitations.	Limits: representativeness, country variation, attrition, ecosystem comparability.
15	Conclusions	Conclusions must align with results.	Outputs: validated drivers, drainers, indicators, and policy levers, generalisable but adaptable.
16	Dissemination	Guidance must be usable and shareable.	Published in journals, policy briefs, SME networks; separate methodological paper prepared.

Table A2. Consolidated Criteria for Reporting Qualitative Studies (COREQ) and Application to Interviews and Focus Groups

No.	Item	Guide Question / Description	Planned Approach for Focus Groups
**Domain 1: Research Team and Reflexivity**			
1	Interviewer / facilitator	Who conducted the focus group?	Research team members at MQU
2	Credentials	Researcher credentials.	PhD students with Master as research
3	Occupation	Researcher occupation.	Economist
4	Gender	Researcher gender.	Male, Female
5	Experience & training	Facilitator training or experience.	3-5 years of research experience
**Relationship with Participants**			
6	Relationship established	Prior relationship?	None expected; recruitment external to research team.
7	Participant knowledge of interviewer	What did participants know?	Informed only of roles and project aims.
8	Interviewer characteristics	Bias, assumptions, interests.	Researchers trained for neutrality; no conflicts of interest.
**Domain 2: Study Design**			
9	Methodological orientation	Analytic framework.	Directed content analysis (with inductive additions)
10	Sampling	Participant selection.	Purposive sampling (entrepreneurs, investors, ecosystem actors).
11	Method of approach	How participants were approached.	Email invitations, LinkedIn outreach, ecosystem contacts.
12	Sample size	Number of participants.	6–8 per focus group.
13	Non-participation	Refusals / dropouts.	To be documented.
14	Setting of data collection	Where collected.	Online via Zoom
15	Presence of non-participants	Anyone else present?	No.
16	Description of sample	Participant characteristics.	Entrepreneurs, startup advisors, accelerator representatives, ecosystem intermediaries.
17	Interview guide	Prompts/questions? Pilot tested?	Semi-structured guide; pilot tested with two advisors.
18	Repeat interviews	Repeat sessions?	No repeat interviews planned.
19	Audio/visual recording	Recording used?	Audio-recorded with consent.
20	Field notes	Field notes taken?	Yes, during and after sessions.
21	Duration	Session length.	50–70 minutes.
22	Data saturation	Was saturation discussed?	Aim for thematic saturation; documented during analysis.
23	Transcripts returned	Returned to participants?	Theme summaries may be provided; raw transcripts not returned.
**Domain 3: Analysis and Findings**			
24	Number of coders	How many coders?	Two
25	Coding tree	Was a coding tree described?	Described
26	Derivation of themes	Predefined or emergent?	Under consideration
27	Software	Software used.	Under consideration
28	Participant checking	Participant feedback?	Under consideration
29	Quotations presented	Participant quotes included?	Yes
30	Data & findings consistent	Were findings consistent with data?	Under consideration
31	Clarity of major themes	Were major themes clear?	Yes; summarised explicitly.
32	Clarity of minor themes	Minority/divergent views included?	Yes; minority perspectives reported.

Appendix B: Data Collection Instruments for Delphi, interviews and focus groups

Table B1. Summary of Delphi Round 1 Questionnaire Items

Section	Content Asked
**1. Background Information**	Role/title; organisation; country; years of experience; education level; sector; type of expertise; gender; email (for linking rounds).
**2. Defining Entrepreneurial Resilience**	Open-ended definition of entrepreneurial resilience, including what should be considered inside and outside the construct.
**3. Drivers and Drainers (Open-Ended)**	List up to three key *drivers* that enhance resilience; list up to three key *drainers* that hinder resilience.
**4. Drivers and Drainers (Literature-Based)**	Review of categories (psychological, social, institutional, strategic/organisational, economic, cultural/gender, digital/AI). Selection of top five drivers from provided list; open-ended additional drivers; selection of top five drainers; open-ended additional drainers; identification of factors that may act as both drivers and drainers.

Table B2. Summary of Delphi Round 2 Questionnaire Items

Section	Content Asked
**1. Rating Drivers**	Participants rate each driver of entrepreneurial resilience on:<br>• Importance (1–5 scale)<br>• Actionability (1–5 scale)<br>• Context sensitivity (COVID, gender, youth, digital, conflict, senior)
**2. Rating Drainers**	Participants rate each drainer of entrepreneurial resilience on:<br>• Importance (1–5 scale)<br>• Actionability (1–5 scale)<br>• Context sensitivity (COVID, gender, youth, digital, conflict, senior)
**3. Additional Suggestions**	Open-ended questions asking whether any additional drivers or drainers should be included.
**4. Final Comments**	Open-ended space for other comments or considerations for subsequent Delphi rounds.

Table B3. Summary of Delphi Round 3 Questionnaire Items

Section	Content Asked
**1. Selecting Measurement Approaches**	Participants choose suitable measurement approaches for each factor (psychometric, longitudinal, theory-driven, econometric, mixed methods). Open-ended space for suggesting additional measurement approaches.
**2. Preferred Outcome Metrics**	Participants select outcome metrics considered appropriate for assessing entrepreneurial resilience (e.g., business survival, growth, adaptability, time-to-recovery, liquidity access, digital capability, workforce resilience). Open-ended space for additional metrics.
**3. Prioritising Metrics**	Participants rank their top three outcome metrics for policy and research prioritisation (Rank 1 = highest priority).
**4. Final Comments**	Open-ended opportunity to provide additional feedback for subsequent Delphi rounds.

Table B4. Summary of Delphi Round 4 Questionnaire Items

Section	Content Asked
**1. Final Consensus on Drivers & Drainers**	Participants indicate agreement (1–5 scale) with the inclusion of each driver and drainer in the final resilience framework.
**2. Prioritising Policy & Program Levers**	Participants rate:<br>• Importance of each policy/program lever (1–5 scale)<br>• Feasibility of implementation (1–5 scale)<br>• Ranking of top three levers (Rank 1–3)
**3. Final Recommendations**	Open-ended feedback on measurement tools, preferred outcome metrics, policy priorities, and strategies for strengthening entrepreneurial resilience.

Table B5. Interview/focus group topic guide

Section	Key Questions / Prompts
**1. Background**	Role; organisation; business stage; sector; years operating; importance of digital tools; exposure to shocks; optional follow-up contact.
**2. Personal and psychological**	Capacities relied on during hardest periods; examples of when confidence or motivation changed; how these shifts affected business decisions.
**3. Social and relational**	Which networks helped most and how they influenced decisions; situations where support was unavailable or insufficient.
**4. Institutional and policy**	Experiences with policies or programs; examples of supportive versus burdensome processes; suggestions for improvement.
**5. Economic and financial**	Role of liquidity, credit, and cash-flow buffers; how financial constraints shaped choices during adversity.
**6. Digital and technological**	How digital tools supported adaptation; examples where digital gaps limited resilience.
**7. Context and time dynamics**	Experiences of repeated or prolonged shocks; how responses changed over time; factors aiding recovery speed.
**8. Driver impact activity**	Card-sorting to rate drivers as high, medium, or low impact and explain why.
**9. Drainer impact activity**	Card-sorting to rate drainers as high, medium, or low impact and explain why.
**10. Support preferences and trade-offs**	Perceived value of different supports (mentoring, grants, digital support, policy fast-tracking); when each is most useful and barriers to access.
**11. Final reflection**	One key recommendation to strengthen entrepreneurial resilience.
