# Supplementary material for: Supporting entrepreneurial resilience: An experimental study protocol
Source: PLoS One. 2026 Jun 4;21(6):e0349194. doi: 10.1371/journal.pone.0349194 (PMC13235934; doi:10.1371/journal.pone.0349194)
Supplement: Attachment — Submitted filename: PLOS1Response to Reviewers.pdf [file pone.0349194.s003.pdf]

Response to Reviewers

Previous Manuscript ID: PONE-D-26-01766

Manuscript Title: Supporting Entrepreneurial Resilience: An Experimental Approach

We thank the PLOS ONE Editorial Office for the guidance on our previous submission. We have carefully revised the manuscript to fully address all concerns and to ensure compliance with PLOS ONE Study Protocol criteria.

Table 1. Point-by-point response

Editorial Comment	Response	Revision Made
Study Protocols should report proposals prior to data collection. The systematic review portion appears completed.	We appreciate this clarification and agree that Study Protocols must focus on prospective research only.	The systematic review is no longer treated as a study component. All methods and findings related to it have been removed. It is mentioned only briefly as background context.
The submission does not meet Study Protocol criteria because part of the study appears completed.	We understand this concern and revised the manuscript to ensure it describes only planned research.	The manuscript now focuses exclusively on the prospective Delphi study, focus groups, interviews, and discrete choice experiment. We clearly state that data collection has not yet commenced.
Consider resubmission if systematic review is removed and focus is on incomplete qualitative work.	We followed this recommendation closely.	The revised manuscript centres on qualitative and experimental components that are fully prospective. The prior review is referenced only as scoping input.
